# A Review on Fucoidan Structure, Extraction Techniques, and Its Role as an Immunomodulatory Agent

**DOI:** 10.3390/md20120755

**Published:** 2022-11-30

**Authors:** Thilina U. Jayawardena, D. P. Nagahawatta, I. P. S. Fernando, Yong-Tae Kim, Jin-Soo Kim, Won-Suk Kim, Jung Suck Lee, You-Jin Jeon

**Affiliations:** 1 Department of Chemistry, Biochemistry and Physics, Université du Québec à Trois-Rivières, Trois-Rivières, QC G8Z 4M3, Canada; 2 Department of Marine Life Sciences, Jeju National University, Jeju 63243, Republic of Korea; 3 Department of Agricultural, Food and Nutritional Science, University of Alberta, 4-10 Ag/For Building, Edmonton, AB T6G 2PG, Canada; 4 Department of Food Science and Biotechnology, Kunsan National University, Gunsan 54150, Republic of Korea; 5 Department of Seafood Science & Technology, Institute of Marine Industry, Gyeongsang National University, Tongyeong 53064, Republic of Korea; 6 Pharmaceutical Engineering, Silla University, Busan 46958, Republic of Korea; 7 Marine Science Institute, Jeju National University, Jeju 63243, Republic of Korea

**Keywords:** fucoidan, anti-inflammation, immunomodulation, structure, extraction, seaweed

## Abstract

Functional ingredients for human health have recently become the focus of research. One such potentially versatile therapeutic component is fucose-containing sulfated polysaccharides (FCSPs), referred to as fucoidans. The exploitation of marine brown algae provides a rich source of FCSPs because of their role as a structural component of the cell wall. Fucoidans are characterized by a sulfated fucose backbone. However, the structural characterization of FCSPs is impeded by their structural diversity, molecular weight, and complexity. The extraction and purification conditions significantly influence the yield and structural alterations. Inflammation is the preliminary response to potentially injurious inducements, and it is of the utmost importance for modulation in the proper direction. Improper manipulation and/or continuous stimuli could have detrimental effects in the long run. The web of immune responses mediated through multiple modulatory/cell signaling components can be addressed through functional ingredients, benefiting patients with no side effects. In this review, we attempted to address the involvement of FCSPs in the stimulation/downregulation of immune response cell signaling. The structural complexity and its foremost influential factor, extraction techniques, have also attracted attention, with concise details on the structural implications of bioactivity.

## 1. Introduction

Marine natural products are widely researched and have produced various promising bioactive components that can be useful functional elements with multiple health benefits [1]. The field of pharmacology has drawn much attention to sulfated polysaccharides purified from brown algae. Recent studies indicate brown algae as a vital and sustainably utilizable industrial source of fucoidans. Structural derivatives of fucose-containing sulfated polysaccharides (FCSPs), such as those originating from Fucales and Laminariales, exhibit anti-inflammatory, immunomodulatory, antiviral, and anticoagulant properties [2,3,4]. Furthermore, the algal source, species, and route of administration contribute to the efficacy of the compound. The bioactivity of purified fucoidans is closely related to their structure. The fucose content, sulfate content, and point of sulfation, including the substitution of monomeric units in the backbone, have been reported to influence bioactivity [2,5]. The complex chemical composition of fucoidans depends on their source, location, and extraction procedure. The intricate structure and molecular weight of fucoidans have hampered the development of standardized fucoidan supplements. Given the correlation between bioactivity and the structure of fucoidans, structural alterations of the target compound should be avoided during purification. Thus, mild conditions are commonly used for the extraction of polysaccharides and isolation of fucoidans [6]. The fine structural elucidation of fucoidans involves mild acid hydrolysis and enzymatic hydrolysis [7,8]. Column purification, multiple chemical modifications, and spectroscopic techniques allow researchers to interpret these complicated structures.

The survival of multicellular organisms largely depends on their capacity to fight infections and recover from injuries. Therefore, the immune system is fundamental. A diverse array of cytokines, signaling proteins, lipid molecules, and transcription factors are involved in immune functions [9]. Complex disorders, such as inflammation, interfere with multiple targets related to drug efficiency and side effects. The pharmacological profile evaluation of natural products has generated profound knowledge, giving rise to drug leads with potentially increased potency on multiple targets [10]. Phytomedicine is defined as the use of plants or herbs for the treatment of human ailments. In this sense, natural products have played a vital role even before written scientific history. Asian countries use seaweed as a common food and medical source. Given its nutrient content, such as dietary fibers, proteins, polysaccharides, vitamins, and minerals, seaweed is widely available and is consumed daily [11]. Diverse cellular and organ functions in multicellular organisms are coordinated by the communication between cells and subcellular compartments. Higher organisms implement multiple mechanisms, including cell signaling molecules, to streamline communication arrays. Kinases, phosphatases, and scaffolding proteins produce specific signals, ultimately leading to chemical modifications. An improved understanding of the cellular signaling pathways and the modulation of particular signal transduction in given cellular systems could assist in the management of diseases [12].

Thus, this review explores FCSPs from marine algae, referring to their structural diversity and extraction techniques accompanied by impurities in isolating standard fucoidan. Furthermore, we reviewed the effects of fucoidans from marine algae on anti-inflammatory and immunomodulatory cell signaling pathways. The influence of the structure on bioactivity was briefly addressed. This review focused on seaweed-extracted fucoidan and its activity. However, fucoidan extraction is not limited to seaweeds from marine resources, and the potential of fucoidans extends beyond the bioactivities addressed herein. Fucoidans have multiple biological benefits that are beyond the scope of this article and are worth addressing in another project.

## 2. Structure of Fucoidan

**Figure 1:**
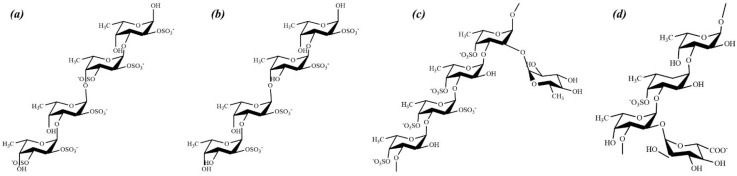
Structural diversity of fucose containing sulfated polysaccharides (FCSPs). Structural models proposed from Fucales, (**a**) *Ascophyllum nodosum* [18], (**b**) *Fucus serratus* [21]. Reported structural models from Laminariales, (**c**) *Chorda filum* [24], and the chemical structural model derived from order Chordariales (**d**) *Cladosiphon okamuranus* [8].

FCSPs are referred to as fucoidans [13]. Fucoidans are polymers in which fucose forms the core monomeric module. The linkage between the monomeric units is either α-(1-2) or α-(1-3). Galactose, mannose, xylose, and glucuronic acid are other possible sugar residues [14]. Acetyl groups and uronic acid are also components of the polymer [13]. The sulfate component is frequently substituted at either the C2 or C4 positions of L-fucopyranosyl residues and randomly at C3 [15]. Its molecular weight distribution is approximately 100–1600 kDa [16]. An analysis of the literature reveals that the fundamental structures of fucoidans from different brown algae species are inconsistent (Figure 1). Categorizing and predicting FCSP structures based on their algal order is paradoxical because of their different compositions. Furthermore, the compositional data vary with the extraction method implemented. Structural analysis of fucoidan involves multistep chemical modifications, such as methylation, desulfation, and vivid spectroscopy techniques (anion-exchange, size-exclusion chromatography, Fourier-transform infrared (FTIR), nuclear magnetic resonance (NMR), and high-performance liquid chromatography–mass spectrometry). Thus, this review discusses data on several FCSPs from brown algae.

Although FCSPs are diverse, their structure has been used to classify them depending on their order to ease the pace of study. The order Fucales has been widely discussed. The backbone structure of FCSP from *Fucus vesiculosus* was presented by Patankar et al. (1993) [17], who proposed that the polymer is primarily composed of α-(1-3) linkages where fucose at branched points is attached via α-(1-2) or α-(1-4) linkages. A significant amount of sulfate substitution was observed at positions 3 and 4, and the fucose core chain was substituted with C-4 sulfates. Chevolot et al. (1999) published research on *Ascophyllum nodosum* suggesting a revised structure for fucoidan [18]. The previous α-(1-2) linked fucose backbone was replaced with alternating α-(1-3) and a high proportion of α-(1-4) linkages. Sulfation was more precisely determined to be *2*-*O*-sulfation and *2*,*3*-*O*-desulfation. Crude fucoidan was extracted using the method described by Mabeau et al. (1990), and acid hydrolysis was conducted using the process described by Colliec et al. (1994) [19,20]. Bilan et al. (2005) anticipated a similar structure. Accordingly, fucoidan from *F. serratus* consists of a backbone with alternating 3- and 4-linked α-fucopyranose residues [21]. β-Xylose and 4-linked α-fucopyranose residues were mentioned as minor chains. The sulfation pattern was determined at positions 2 and 4, and some terminal residues remained non-sulfated. The polymer was also supported by xylose, mannose, glucose, and uronic acid, in addition to the major monomeric units. Fucoidan was isolated by collecting *F. serratus*, performing air and vacuum drying, and then milling the algal biomass at room temperature with a 4:2:1 MeOH:CHCl_3_:H_2_O mixture to eliminate the color compounds in the structure. Then, the material was mechanically stirred with CaCl_2_, and the crude polysaccharide was obtained through centrifugation, combined, dialyzed, and lyophilized to form a mixture consisting of 32.8% fucose, 24.4% glucose, 18.9% SO_3_Na, 2.9% xylose, 2.6% galactose, and 1.8% mannose. Column fractionation was performed on the above sample containing DEAE-Sephacel (Pharmacia) in Cl^−^ form, with gradient concentrations of NaCl. Ion-exchange chromatography produced four different fractions with considerable yields [22]. *Sargassum stenophyllum* was reported to have two types of galactofucans, which resembled the fucosylated chondroitin sulfates from sea cucumbers [23]. They were composed of a linear core and branched chains of fucans. The core chain was formed by (1-6)-β-D-galactose and/or (1-2)-β-D-mannose units. The branched chains consisted of 3- and 4-linked α-L-fucose, 4-linked-α-D-glucuronic acid, β-D-xylose, and 4-linked-α-D-glucose. Two of the identified fucans had different structures depending on their composition. Type I was abundant in glucuronic acid and contained fewer sulfate groups, whereas type II included xylose, mannose, galactose, glucose, and uronic acid, in addition to the general chemical composition. During the isolation process, crude polysaccharides were precipitated with ethanol three times, treated with CaCl_2_, and further precipitated with cetylpyridinium chloride to obtain a higher yield of fucoidan fractions. CaCl_2_ was used to dissolve the pyridinium salts and reprecipitation was performed using ethanol. Moreover, insoluble and soluble fractions were obtained via the centrifugation of ethanol-insoluble material dissolved in water. Another study focused on the order Fucales using *S. polycystum* and revealed a novel sulfated galactofucan. This polymer had a backbone of 3-linked α-L-fucopyranose and 4-linked sulfation. Furthermore, the backbone contained random branches of 2-linked α-D-galactopyranose, which were sulfated at position 4.

The brown seaweed *Chorda filum* was assessed for its fucoidan fractions [24]. Its chemical composition was reported to include fucose, xylose, mannose, glucose, galactose, uronic acid, and sulfate. Structural analysis revealed the presence of an α-(1-3)-linked backbone with multiple branching and a majority of α-(1-2)-linked monomers. Sulfation was mainly observed in position 4 and rarely in position 2. Furthermore, *2*-*O*-acetylated random α-(1-3)-linked fucose residues have been reported. Similar to fucoidan isolated from the previously mentioned species, fucoidan fractions have been obtained via ion-exchange chromatography using DEAE Sephadex A-25. Structural analysis of fucoidans is prevalent for the order Fucales but has also been reported for the order Laminariales. Wang et al. (2010) obtained three fractions of low-molecular-weight fucogalactan from *Laminaria japonica* [25]. The prominent fraction was the second, which showed that 75% comprised 3-linked α-L-fucopyranose, and the remaining fraction was the 4-linked α-L-fucopyranose residues. The researchers recorded multiple branching points indicating a 65% molar ratio with non-reducing terminal fucose at C-2 of α-(1-3)-L-fucopyranoside residues. The sulfation points were recorded with variations. Fucoidan was isolated from *L. japonica* using water and further purified through ethanol precipitation. Usoltseva et al. (2019) assessed purified sulfated fucans from *L. longipes* and *Saccharina cichorioides* (Laminariales) [26]. Accordingly, the fucan structure mainly consisted of (1-3), (1-4), and (1-2)-linked α-L-fucopyranose units. Sulfation was present at positions 2 and 4 of the respective (1-3)- and (1-2)-linked α-L-fucopyranose units. During extraction from the brown alga *S. cichorioides*, the sampling material can be reduced to a practical size of 1 mm; ethanol, acetone, and chloroform were added to increase the efficiency of the extraction process. Fat was removed from the sample, followed by the addition of 0.1 N HCl (2 L) at room temperature. Ultrafiltration was performed to concentrate the sample to up to 20% of the initial volume, and the sample was washed four times with 96% ethanol for the further precipitation of polysaccharides. Subsequently, 96% ethanol was used to wash the pellet, and air-dried samples were used in further experiments. A DEAE-cellulose column was used for the fractionation of fucoidan [27]. Other algal orders have been reported for fucoidan structures. Similar to those in the order Laminariales, these structures were substituted with monosaccharides other than fucopyranose. Among them, *Cladosiphon okamuranus* from the order Chordariales showed a linear backbone of α-(1-3)-fucopyranose, half of which was sulfate substituted at the C4 position. Some fucopyranoses were *O*-acetylated [8]. Backbone fucose was substituted by C2 α-glucuronic acid. The C2 α-glucuronic acid-substituted fucose units were not sulfated at C4, and its chemical composition has been reported to include fucose, glucose, uronic acid, and sulfate. The extraction and isolation of fucoidan have been described by Masato Nagaoka et al. (1999) [8].

It is evident that the structural composition of fucoidans varies among different brown seaweed species. We have elaborated on multiple structures discussed in previous literature depending on seaweed order, but neither of those supports a consistent basic structure of fucoidan. This proves that the structural traits of FCSPs cannot be predicted or categorized due to their complexities among each other. Though the chemical composition of fucoidans is complex, the basic substituents are fucose and sulfate. Moreover, the fucoidans are consisted of other monosaccharides, uronic acid, and protein. Other than these the structural features will be determined by age, geographic region, and the harvesting season. A previous study reported that the fucose content increased during the sporulation period in *F. vesiculosus* compared with other periods. They also reported that the fucose content was lower in the sterile phase than in the fertile phase [28]. Moreover, in Far Eastern brown macroalgae, sporulation occurred when most polysaccharides accumulated, which was followed by a change in the monosaccharide composition [29]. Another study demonstrated that *L. japonica* showed increased levels of fucose content during the reproductive phase [30].

## 3. Extraction Techniques and Potential Impurities

The nature of the chemical components, method of extraction, and interfering substances influence the extraction [31]. Most polysaccharides are extracted using water, acids, and organic solvents. Solvent extraction is difficult to implement owing to the complex nature of polymers [1]. The extraction procedure is a critical point in the purification of the target compound. The yield and structural alterations of sulfated polysaccharides are greatly influenced by the appropriate adjustment of parameters such as temperature, time, and pH [6]. This part of the review addresses the implemented extraction procedures that effectively enhance the purification of sulfated polysaccharides.

**Figure 2:**
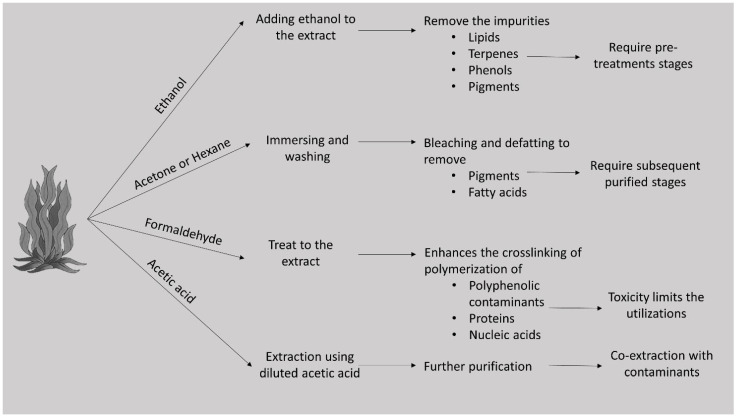
Effect of solvents on extraction/purification of FCSPs.

The source of extraction of the sulfated polysaccharides is marine brown algae rather than marine invertebrates. The molecular weight, sulfation percentage, point of sulfation, and monosaccharide composition are vital to the bioactivity of fucoidan. Therefore, purification should be performed under mild conditions to avoid structural shifts [6]. The application of an ethanolic system helps remove co-extracting interferences, such as lipids, terpenes, and phenols (Figure 2). Chlorophyll pigments are immediately removed using this method. Some researchers perform immersion and washing using acetone or hexane. Phenols are tightly bound to fucoidans or other polysaccharides during the extraction process. Formaldehyde treatment is a method of preventing cross-contamination by polymerizing phenols, proteins, and nucleic acids, and converting them into insoluble high-molecular-weight components [6,32,33]. Although it is useful in eradicating interfering modules, it may decrease fucoidan yield by interacting with polysaccharides and precipitating them into complexes [34].

Fucoidan extraction using dilute acetic acid has a history of over 100 years, with written scientific records. In 1913, Kylin termed these compounds fucoidan [35]. Currently, this term has been modified to FCSPs. Although FCSPs were reported to contain fucose as the main constituent and mannitol, alginic acid, and laminarin as co-existing components, it is now known that the latter are contaminants. Alginate contamination has been widely observed. Nelson and Cretcher reported that fucose is the only sugar present in the unhydrolyzed seaweed *Macrocystis pyrifera*, in which hydrolysis results in uronic acid [36]. The removal of soluble alginates via their conversion into insoluble calcium salts is a useful technique in this regard. Proteolytic enzyme practices facilitate the removal of protein contamination. Multiple reports published by Jayawardena et al. in recent years have suggested a modified method to purify FCSPs, implementing methods for the removal of alginate and protein contamination [5,13]. In acid-treated fucoidan extraction, the acid concentration, time, and temperature of extraction are vital factors influencing the sample yield, sulfate, and fucose content. Higher acid concentrations lead to cell wall matrix penetration and disruption of the fucoidan structure. A longer extraction time decreases the fucose and sulfate contents, resulting in low-quality fucoidan [23,37].

Purification of high-quality fucoidan requires further steps assisted by column chromatography. One such method is anion-exchange chromatography. The sulfate ester groups linked to the polymer backbone provide anionic charges even at low pH values, which can be utilized during this chromatographic technique. The resin used in this method is quaternary ammonium, which forms strong coulombic interactions with the loaded sample. Elution is performed with the application of a NaCl gradient, where its concentration increases at the later stages of the column purification mobile system. The elution of lower sulfate-containing fractions occurs first, whereas the high sulfate fractions elute last [13,38]. The fractions can be further subjected to gel permeation chromatography to remove salts. Dialysis membranes have been reported to assist further purification; specifically, the semipermeable membranes diffuse small impurities and contribute to de-salting [7,39].

Jasso et al. (2011) implemented microwave-assisted extraction (MAE) with optimum reaction conditions to extract sulfated polysaccharides from *F. vesiculosus* [40]. They summarized it as an effective method that requires a shorter extraction time, has reduced costs, and consumes less energy. Moreover, this method generates less waste, thus proving to be environmentally friendly. However, it is highly recommended to manage and optimize the energy input. This technique distributes a heat source owing to the friction generated from the dipolar rotation of polar solvents and the conductive migration of dissolved ions [41,42]. This wields pressure on the cell wall, resulting in cell wall breakage and the release of intracellular components. Another approach is ultrasound-assisted extraction (UAE), which is based on cavitation generation via ultrasound waves. Turbulence and interparticle collisions convert sound waves into mechanical energy, which disrupts the cell wall. Fernandez et al. (2016) reported the use of UAE to extract fucoidan from *S. muticum* [43]. Fucoidan from *F. evanescens* was extracted using the method described by Hmelkov et al. (2017) [44]. Similar to MAE, the application of energy should be optimized to avoid any possible structural alterations.

The efficient extraction of seaweed polysaccharides is hindered by the complexity and rigidity of the cell wall. This has led to the use of carbohydrates to degrade the cell wall, thus releasing polysaccharides and other bioactive components. Each carbohydrase must be implemented at its optimal pH and temperature to obtain higher yields with promising bioactive properties [45]. Sanjeewa et al. (2017) isolated sulfated polysaccharides from *S. horneri* using AMG, Celluclast, Viscozyme, and Alcalase. The chemical properties of each isolate were evaluated for their anti-inflammatory potential [46]. The extraction methodologies involved have a major influence on the structure, composition, and yield of fucoidans, and the biological activity of the isolated sulfated polysaccharides is evaluated. According to a recently published study, the anti-inflammatory effects of fucoidan are strongly, moderately, and weakly correlated with the fucose, sulfate, and xylose contents, respectively. In addition, the antioxidative activity is positively correlated with the total polyphenol content [47].

Availability, suitable extraction methods, algal sources, yield, and purity should be considered for mass production. A suitable procedure for commercial fucoidan production was published in 1952 [32]. Recently, this procedure was adjusted to production requirements. Numerous modifications of the isolation procedure were aimed at accelerating the process or increasing the yield of target polysaccharides, as well as improving their purity [48]. Downstream processing is a vital step in maintaining the quality of the ultimate product. Therefore, the removal of impurities, including proteins, pigments, and biopolymers, is necessary for commercializing fucoidan products [49]. The most common sources used to produce commercial fucoidans are *F. vesiculosus* and *A. nodosum* which are Atlantic representatives of Phaeophyceae and exhibit similar chemical composition and biological properties [48]. In addition to these common species, numerous studies are ongoing to commercialize high-value products.

## 4. Immunomodulatory Effects

Inflammation can arise in any tissue in response to multiple conditions, such as infectious, toxic, or autoimmune injury. Inflammation does not reflect a single, straightforward mechanism but is a complex set of axes that interact to overcome detrimental situations. However, if the situation is not properly handled through this multifaceted mechanism, it can lead to persistent tissue damage via the recruitment of cellular members of the immune system. The neutral status of inflammation does not arise passively and exhibits proper maintenance of health requirements by ample positive actions where it does not entail activation of inflammation. Modulation of inflammation is an alternative approach, although pathogenic events are unknown [50]. However, in severe situations, the inflammatory response may cause extensive damage compared with the source of infection. Intrinsically, this has become a reason to target inflammation as a therapeutic cross point in a range of disorders. Although research regarding anti-inflammation occupies a wide share of publications, its corresponding importance is attributed to a sufficient review of the promotion of inflammation. This is because inflammation provides immunization against a broad range of infections. Cancer immunotherapy considers immunostimulation to be a critical step in preventing tumor recurrence [51].

Discussions regarding the health benefits of food beyond basic nutrition have increased public awareness in recent years. The health-promoting effects of foods and the implementation of these bulk sources as functional foods have become a widely researched topic. Thus, significant developments in the natural products of nutraceuticals, pharmaceuticals, and functional foods have opened up broader topics in the research community [1]. The potential bioactivities of fucoidan (FCSPs) from seaweeds have been assessed. This section of the review emphasizes the immunomodulatory (pro- and anti-inflammatory) potential of FCSPs (Table 1 and Table 2), as well as the structure–activity relationship.

Kim et al. (2012) reported that fucoidan isolated from *U. pinnatifida* intervened in obesity-associated inflammation-related metabolic syndrome [52]. Inflammation-related cytokines, namely tumor necrosis factor-α (TNF-α), monocyte chemotactic protein-1 (MCP-1), and plasminogen activator inhibitor-1 (PAI-1), were assessed for their involvement in adipocyte inflammation, and thus in the development of adipogenesis. Fucoidan from *U. pinnatifida* downregulated the above inflammatory factors, resulting in the inhibition of adipogenesis. Early reports explained that TNF-α regulates both MCP-1 and PAI-1 [53]. The analyzed fucoidan consisted of 25% organic sulfates and 60% total carbohydrates, with a protein content as low as 5% [52]. The high sulfate content of crude fucoidan may have contributed to its bioactivity, although the authors did not discuss this matter in detail. A recent publication by Fernando et al. (2021) suggested low-molecular-weight fucoidan as an ailment against fine-dust-stimulated inflammation in keratinocytes [2]. *S. horneri* was used to isolate low-molecular-weight fucoidan. The FTIR and NMR results collectively signify the structural similarity to commercial fucoidan. The chemical composition of the low-molecular-weight (~45 kDa) fractions (SHC4–6) reveals the structure–activity relationship bearing 43.15 ± 0.36% polysaccharide and 29.92 ± 0.29% sulfate levels. Polyphenol and protein interferences were negligible, and fucose was as high as 36.50 ± 1.45%. The authors discussed the involvement of the nuclear factor kappa B (NF-κB) and mitogen-activated protein kinase (MAPK) pathways in the downregulation of inflammation caused by fine dust.

Regulation of the immune response gene expression is critical for the maintenance of immune homeostasis. One of the most significant effector pathways involved in inflammation is NF-κB, which is a transcriptional regulator composed of Rel-protein homo- or heterodimers [54]. This regulator is found in the cytoplasm of quiescent cells and binds to the IκB inhibitor, blocking its nuclear localization. When IκB is active, it is phosphorylated and released from the complex, and the Rel-proteins enter the nucleus and selectively bind consensus DNA elements to initiate the activation of a cascade of inflammatory-related genes, and hence, the creation of different mediators [54]. Mammals possess five types of proteins with RHD: p65 (RelA), RelB, c-Rel, p100/p50, and p105/p52. In the unstimulated status, NF-κB is associated with IκB proteins (IκBα, IκBβ, IκBε) and is retained in the cytoplasm, thereby preventing the nuclear translocation and subsequent DNA binding of p50:p65 heterodimers [55]. IκB proteins are characterized by the presence of multiple ankyrin repeat domains [56]. Upon stimulation, the IκB kinase complex is activated and degrades IκB, drastically altering the dynamic balance of NF-κB dimers and favoring its nuclear localization. However, the representation of proteins involved in the process varies depending on whether the route taken is canonical or non-canonical. A detailed review of the subject matter was published by Hayden and Ghosh, who provided illustrations of the shared principles in NF-κB signaling [57]. The underlying mechanism of many fucoidans is the inhibition of the generation of pro-inflammatory cytokines by decreasing NF-κB activation. Fucoidans exhibit anti-inflammatory activities by modulating the NF-κB pathway. Sanjeewa et al. used Celluclast-assisted digestion followed by ultrafiltration to extract four fucose-rich sulfated polysaccharide fractions with different molecular weights from *S. horneri* [58]. Among these fractions, the one with a molecular weight greater than 30 kDa (F4) inhibited lipopolysaccharide (LPS)-induced the production of NO (IC50 = 87.12 g/mL), PGE_2_, and pro-inflammatory cytokines in RAW 264.7 cells via downregulating the NF-κB signaling cascade [58]. Researchers have also shown that the levels of iNOS and COX-2 inhibition caused by F4 at concentrations of 100 g/mL and 50 g/mL were equivalent to those caused by aspirin at 12.5 g/mL. F4 also suppressed LPS-induced toxicity, cell death, and NO generation in an in vivo zebrafish embryo model [58]. Pozharitskaya et al. (2020) discussed in detail the correlation of anti-inflammatory, fucoidan, and COX activities in a review article [59].

Another pathway that has been subjected to broader discussion involves MAPKs. This protein structure is well-conserved from unicellular eukaryotes to multicellular organisms. Specifically, this structure includes three proteins, i.e., extracellular signal-regulated kinase (ERK), c-Jun N-terminal kinase (JNK), and p38, which sequentially phosphorylate to initiate the signaling cascade. MAPKs are not only involved in the regulation of inflammation but also play an important role in cell survival, proliferation, differentiation, and death. Huang et al. published a detailed review of each MAPK component [60].

Jayawardena et al. (2020) analyzed fucoidan derived from *S. swartzii* with a high sulfate content against LPS-stimulated inflammatory responses in RAW 264.7 macrophages [5]. They reported a full array of signaling cascades starting from toll-like receptors (TLRs) down to NF-κB and MAPK, as well as the production of cytokines and inflammatory modulators such as NO. The sulfate content in the active fucoidan fraction of *S. swartzii* was 33.99 ± 0.17%. Although the authors did not propose a detailed structure of the isolated fucoidan, the chemical composition showed a higher sulfate content and fucose monosugar. Therefore, the active fraction may have influenced the results. According to this report, canonical p65 nuclear translocation was evident using immunofluorescence techniques. TLRs, which are located in the cytoplasmic membranes of macrophages, share a toll-interleukin receptor domain. These signals are cascaded from MyD88, IL-1 receptor-associated kinases (IRAKs) to NF-κB [61]. Sanjeewa et al. (2019) published a similar report, evaluating fucoidan isolated from *Padina commersonii* [38]. TLR2/4 was assessed and mentioned to be the trigger point of LPS-driven signals to induce the NF-κB pathway. Accordingly, TLR2/4 and MyD88 were found to be vital in LPS-driven inflammation, and fucoidan from *P. commersonii* significantly inhibited their expression, altering downstream signal transduction. Another study on fucoidan derived from the invasive species *S. horneri* reported the inhibition of LPS-induced inflammation via the NF-κB and MAPK pathways [62]. The phosphorylation of MAPKs (p38 and ERK1/2) was significantly inhibited by fucoidan co-treatment. Thus, the production and expression levels of NO, PGE_2,_ and pro-inflammatory cytokines (TNF-α and IL-6) decreased [13,55].

MAPKs are key players in inflammatory processes, such as upstream NF-κB signaling molecules [63]. Wu et al. found that fucoidan from the brown alga *S. cristaefolium*, in addition to reducing NF-κB activation, dramatically reduced the production of iNOS by decreasing the phosphorylation of p38, ERK, and JNK in LPS-stimulated RAW 264.7 cells [64]. They also discovered that fucoidan from the same source, but with varying molecular weights or sulfate concentrations displayed distinct anti-inflammatory effects. Zhang et al. discovered that the inhibition of the TLR4-MAPK-NF-κB signaling pathway via oral administration of polysaccharides from the Seabuckthorn berry for 14 consecutive days significantly lowered the expression of TNF-α, IL-1β, and iNOS in the serum of CCl4-challenged mice [65]. Another study showed that sulfated polysaccharides extracted from *S. horneri*, a brown seaweed, had anti-inflammatory properties [46]. These enzymes included AMG, Celluclast, Viscozyme, and Alcalase. In LPS-stimulated RAW 264.7 cells, researchers discovered that fucoidan from Celluclast enzyme digest (CCP) demonstrated the strongest suppression of NO production and downregulated the expression of iNOS and COX-2, as well as the release of TNF-α and IL-1β. Further research revealed that fucoidan exerted its anti-inflammatory effects by preventing MAPK and NF-κB activation by decreasing the phosphorylation of p38 and ERK, as well as the nuclear translocation of NF-κB subunits p50 and p65.

NO Is used as a primary evaluation cross-point in inflammation studies. It is a vital end product and is regulated by iNOS. Mammalian cells are capable of producing three forms of nitric oxide synthase (NOS) depending on the cell type: iNOS, eNOS, and nNOS, where l-arginine acts as the primary substrate. NO production exceeds regular levels in the presence of immunological reactions; thus, it is involved in pathogenesis [66]. The results of the aforementioned studies [2,5,38,62], showed that NO levels were elevated via LPS stimulation and affected neighboring cells, as explained by cell viability. However, the isolated fucoidan fractions (the active fraction in each study) successfully inhibited the negative impact of restoring cell viability and reducing NO production. COX-2 is another assessed inflammatory modulator. COX-2 is a heme-containing glycoprotein present in two isoforms; COX-1 and COX-2, where COX-2 is the primary enzyme controlling the synthesis of PGE_2_ via the arachidonic acid pathway. Thus, the modulation of COX-2 is as important as that of iNOS in inflammatory pathogenesis [67]. Inflammatory cytokines are another vital factor coordinating inflammation. Inflammatory cytokines are vital factors that coordinate inflammation, as reported by Nathan (1987) [68]. Cytokines are defined as lycolo)proteins released via living cells and exhibit a non-enzymatic mode of action at lower concentrations, such as pico/nanomolar, to regulate proper cellular function [68]. Therefore, studies on inflammation have used these mediators as targets for evaluation.

The Janus kinase (JAK)-signal transducer of activators of transcription (STAT) pathway is another vital pathway associated with diverse cytokines, interferons, growth factors, and related molecules. Receptor-associated JAKs are activated by ligand binding to cognate receptors. Successive STAT activation conveys rapid signaling from the cell surface to the nucleus, allowing subsequent translocation of STATs to the nucleus, where they modulate target gene expression [69]. Ye et al. (2020) studied the fucoidan active fraction (SF6) from *S. japonica* against LPS-induced inflammation in macrophages [70]. This report explains the potential of SF6 to significantly regulate JAK2 phosphorylation and downregulate its associated STAT1/3. Thus, the expression of pro-inflammatory cytokines decreased. LPS, an endotoxin, substantially promoted the phosphorylation of JAK2 and STAT1/3 compared with the control group. The authors suggested that SF6 might target JAK2 and subsequently STAT1/3 to exert its anti-inflammatory effects.

Immunomodulation is the navigation of the immune defense system via medication. Immune response enhancement has been reported for many polysaccharides obtained from natural resources [71]. Yang et al. (2008) reported significant collaboration to promote dendritic cell (DC) maturation using fucoidan from *F. vesiculosus* [72]. This process was simultaneous with cytokine- and T-cell-mediated immune responses. Cell differentiation was driven by the Th1-polarizing phenotype upon fucoidan stimulation. Subsequently, TNF-α and IL-12 secretion enhanced immune capacity. Researchers have suggested fucoidan in the application of DC-based vaccines for cancer immunotherapy. A similar study by Kim and Joo (2008) revealed that the translocation of p65 to the nucleus in DCs was promoted by fucoidan from *F. vesiculosus* [73], providing evidence for the involvement of the NF-κB pathway in immunostimulation. The effects of fucoidan from *F. vesiculosus* were investigated in spleen DCs [74], and the expression of CD40, CD80, and CD86 with TNF-α, IL-6, and IL-12 was found to be promoted. Fucoidan treatment induced the IL-12-dependent generation of IFN-γ-producing Th1 and Th2 cells. Two types of macrophages were assessed for fucoidan from *U. pinnatifida*. RAW 264.7 macrophages expressed chemokines (RANTES and MIP-1α), whereas murine splenocytes produced IL-6 other than chemokines [75]. Do et al. (2010) studied the IFN-γ-induced production of NO and its underlying mechanisms, involving the effects of fucoidan from *F. vesiculosus* [76]. This report published data on glia and macrophages. The results showed contrasting expression levels of fucoidan in response to IFN-γ stimulation. The NO/iNOS produced by the stimulation of IFN-γ in glial cells was inhibited by fucoidan. The action of fucoidan was evident through the suppression of JAK/STAT/IRF-1 and p38 activation. Conversely, in RAW 264.7 cells, the expression of iNOS induced by IFN-γ was further stimulated by fucoidan. In RAW macrophages, TNF-α stimulation and p38 activation are the cross-points of fucoidan action. This research shed light on the action of p38 depending on the cell type, where fucoidan was proposed as an immunomodulatory nutrient with alternating sensitivity to p38 activation.

The anti-inflammatory and immunomodulatory activities of fucoidan are solidified not only in vitro but also in vivo. Previous studies have revealed that oral administration of these polysaccharides results in low levels in the plasma and high levels in particular organs; for example, fucoidan isolated from *C. okamuranus* accumulated more in the liver of rats than in their blood serum. Furthermore, the transport mechanism of fucoidan was identified, and active transport through the cell membrane was suggested as the most probable mechanism [77,78,79]. The stability of the compound in the in vivo model is also an important factor in evaluating its anti-inflammatory or immunomodulatory activity. The administration method is a major factor that determines the half-life of fucoidan in an in vivo model. This was confirmed by a previous study, which reported that fucoidan isolated from *F. vesiculosus* exhibited superior skin penetration by topical application in rats and that a 100 mg/kg dose showed a prolonged half-life compared to that of intravenous injection [80]. The anti-inflammatory activity of fucoidan isolated from *S. japonica* has been reported to reduce inflammation, oxidative stress, hepatorenal injuries, and DNA damage via the downregulation of pro-inflammatory cytokines such as IL-1β, IL-6, and TNF-α in streptozotocin-induced diabetes mellitus in rats [81]. A similar study was published by Xu et al. (2017), who showed a reduction in IL-6 in the kidneys after treatment with low-molecular-weight fucoidan [82]. Furthermore, the treatment of diabetic mice with low-molecular-weight fucoidan combined with fucoidan isolated from *S. hemiphyllum* significantly reduced the TNF-α levels [83]. Matsumoto et al. (2004) studied the effect of fucoidan isolated from different seaweeds in a chronic colitis mouse model and revealed a decreasing effect on IFN-γ and IL-6. However, this study showed that fucoidan extracted from *F. vesiculosus* did not have a significant effect in this in vivo model [84]. However, another study reported that fucoidan from *F. vesiculosus* had a significant downregulatory effect on IL-10, IL-1β, and IL-1α in a dextran sulfate sodium-induced mouse model of acute colitis [85]. In anti-inflammatory studies, zebrafish models are frequently used as in vivo models in human disease studies. According to a recently published study, fucoidan isolated from *Chnoospora minima*, *Ecklonia cava*, and *S. horneri* decreases the production of COX-2 and NO in LPS-induced zebrafish embryos [7,58,86]. Low-molecular-weight fucoidan isolated from *U. pinnatifida* showed significantly reduced tissue infiltration with inflammatory cells and bone and cartilage destruction in a rheumatoid-arthritis mouse model [87]. Fucoidan isolated from *U. pinnatifida* and *Turbinaria ornate* downregulated arthritis in rats induced by Freund’s adjuvant by decreasing the levels of PGE_2_, TNF-α, and IL-6 in blood plasma [88,89]. Fucoidan from *T. decurrens* significantly reduced the expression of IL-1 β and COX-2 in a formalin-induced edema mouse model. AlKahtane et al. (2020) performed an anti-inflammatory study of *L. japonica* fucoidan against microcystin-LR-induced liver damage and found a significant reduction in the levels of IL-6, TNF-α, and IL-1β in the serum of mice [90]. Chale-Dzul et al. (2020) identified the anti-inflammatory effect of fucoidan isolated from *S. fluitans* against carbon tetrachloride-induced liver injury in rats. The results of this study confirmed a significant reduction in the levels of IL- 1β and TNF-α [91]. In addition to these anti-inflammatory studies, a study focused on the effect of fucoidan from *F. vesiculosus* on an LPS- and *Porphyromonas gingivalis*-injected periodontitis mouse model, which acted by downregulating IFN-γ [92]. Furthermore, neutrophil infiltration of the peritoneal cavity of an acute-peritonitis rat model was regulated by *C. okamuranus* fucoidan by decreasing the pro-inflammatory cytokine levels, such as TNF-α, IL-1β, and IL-6, and downregulating NF-κB signaling [93].

The anti-inflammatory activity of fucoidan has gained much attention, and studies have shown its immunomodulatory potential. Some studies have revealed that low-molecular-weight fucoidan has anti-inflammatory activity and high-molecular-weight fucoidan exhibits immunomodulatory activity [87]. However, this is not a consistent phenomenon; both low- and high-molecular-weight fucoidan from some species shows anti-inflammatory effects [46,94]. Fucoidan isolated from *U. pinnatifida* significantly increased the levels of natural killer cells, neutrophils, and pro-inflammatory cytokines in the body and downregulated apoptosis. *F. vesiculosus* fucoidan also elevated DC maturation, Th1 immune responses, and cytotoxic T-cell activation, and increased the production of antibodies after exposure to antigens [74]. Furthermore, fucoidan purified from *F. evanescens*, *L. cichorioides*, and *L. japonica* has been shown to activate the immune system. Makarenkova et al. (2012) demonstrated that the interaction between fucoidan and TLRs increases the production of cytokines and chemokines. According to these results, fucoidan binds to TLRs and enhances NF-κB signaling; this results in the production of cytokines and inflammatory responses [95]. Hayashi et al. (2008) reported that natural killer cells and augmented T cells were activated by treatment with fucoidan isolated from *U. pinnatifida*. Furthermore, this treatment elevated the production of pro-inflammatory cytokines in a herpes simplex virus (HSV-1)-infected mouse model [3]. Jang et al. (2014) performed a comparative study of the immunomodulatory activity of fucoidan purified from four seaweed species, i.e., *F. vesiculosus*, *U. pinnatifida*, *A. nodosum*, and *M. pyrifera*, revealing significant upregulation of IL-8, IL-6, and TNF-α production. *M. pyrifera* showed superior activity among these seaweeds [96]. Fucoidan isolated from *U. pinnatifida* increased IFN-γ production in a UVB-induced mouse model. However, this study reported that it did not increase cytokine production but reduced edema and leukocyte migration to the skin [97].

In addition to these in vitro and in vivo evaluations, clinical trials have been reported by Myers et al. (2016). According to the results of this study, patients (seven males and five females) with osteoarthritis were treated with fucoidan. In brief, five participants were treated with 100 mg/day and seven participants were treated with 1000 mg/day of Maritech^®^. This was a combination of *M. pyrifera* (10% *w/w*), *F. vesiculosus* (85% *w/w*), *L. japonica* (5% *w/w*), zinc, vitamin B6, and manganese. The results showed an overall reduction in symptoms such as stiffness and pain during physical activities. Furthermore, a 1000 mg/day dose showed prominent results [98]. Nagamine et al. (2020) also evaluated the immunomodulatory activity in clinical trials. This study reported the effect of fucoidan purified from *C. okamuranus* on the activation of natural killer cells in cancer survivors. The results suggested that 3 g of fucoidan was a safe dose for patients, and recurrence of tumors significantly decreased [99].

Hayashi et al. (2008) assessed the extended scope of fucoidan overstimulation of both innate and adaptive immune defense functions, revealing the potential of fucoidan to inhibit viral replication [3]. Fucoidan from *U. pinnatifida* was orally supplemented to BALB/c mice, and its effects on viral replication in the HSV-1-infected in vivo model were assessed. Oral supplementation of fucoidan was determined to protect the mice against viral infection based on the survival rate and lesion scores. Fucoidan administration enhanced cytotoxic T lymphocyte activity. In vitro assays confirmed improvements in phagocytic activity and B-cell blastogenesis upon treatment with fucoidan. In conclusion, this study suggested that the effect of orally administered fucoidan largely depends on gut immunity in animals. Tabarsa et al. (2020) reported the immune-boosting properties of fucoidan from *Nizamuddinia zanardinii* [100]. This action is evident through the activation of macrophages and NK cells. The immunoenhancing properties of the active fraction were attributed to the highly branched C2 sulfated fucose backbone and galactose residues. Immune activation occurs through the NF-κB and MAPK pathways.

Owing to their low toxicity and great oral bioavailability, fucoidan and fucoidan-derived products have recently been commercialized in different sectors, including cosmetics, dietary supplements, and animal-feeding supplements [79]. Fucoidan-derived products have gained regulatory approval in several global jurisdictions. The United States Food and Drug Administration has approved fucoidan extracts from *Undaria pinnatifida* and *F. vesiculosus* by the Australian manufacturer Marinova as “Generally Recognized as Safe (GRAS)”. The European Commission approved fucoidan extracts from the same species as a novel food [101]; respective agencies from Canada and Australia have also approved numerous listed fucoidan-containing medicines. Fucoidan was approved for oral consumption of up to 250 mg/day under the Commission Implementing Regulation of European Union 2017/2470 on 20 December 2017 [102].

Despite the use of fucoidan and fucoidan-derived products as food and dietary supplements, there are several examples of the combination of fucoidan with clinical, chemical, and chemotherapeutic agents as combination therapy. In Taiwan, a human clinical study of lung cancer patients showed that treatment with cisplatin and fucoidan efficiently increased the survival rate of patients [103]. Studies indicate the potential of the combination of fucoidan and cisplatin for preventing human lung tumorigenesis as an effective therapeutic agent. A study in Australia examined the effect of the co-administration of fucoidan derived from *U. pinnatifida* on letrozole, tamoxifen, and hormone-treated breast cancer patients. This study showed no significant difference in the risk of clinically significant interactions and adverse effects of fucoidan treatment [104]. A prospective, randomized, double-blind, controlled clinical trial with metastatic colorectal cancer (mCRC) patients was conducted between 2014 and 2016 in southern Taiwan. The patients were administered a first-line chemo-target regimen using folinic acid, 5-fluorouracil, and irinotecan (FOLFIRI) plus bevacizumab (Avastin^®^ injection) along with fucoidan powder bis in die (twice a day in Latin); the administration of Taiwan oligo fucoidan^®^ effectively increased the disease control rate of mCRC patients [105]. The results of clinical trials in humans demonstrate the potential of fucoidan as an adjuvant therapy for cancer treatment and its further development into a commercialized drug. For cardiovascular diseases, a new fucoidan-based pharmaceutical product was developed after the evaluation of its human safety [106].

**Table 1:** Anti-inflammatory potential of fucoidan purified from marine algae.

Study	Cell Line	Concentration	Algal Species and Fucoidan Purified	Cell Signaling Activity	Reference
Inflammatory related cytokine modulation and expressing anti-obesity effects	3T3-L1	1–100 µg/mL	*Undaria pinnatifida* Polysaccharides (≈60%) and sulfate (≈25%) with less protein content	Significantly decrease the expression of inflammation-related genes during adipogenesis in 3T3-L1 adipocytes. Adipogenesis major markers (c/EBPα, PPARγ) were down regulated via fucoidan. Inactivation of aP2 led to the weakening of TNF-α, MCP-1, PA-1 levels. Lipid accumulation and ROS content in adipocytes were attenuated by fucoidan.	Kim et al. (2012) [52]
Fine dust (FD) induced inflammatory responses in HaCaT keratinocytes are ameliorated by fucoidan	Human skin keratinocytes (HaCaT)	12.5–100 µg/mL	*Sargassum horneri* purified fucoidan fraction SHC4-6 was reported as a highly sulfated mannofucan (≈45 kDa)	SHC4-6 dose-dependently lowered ROS levels in Fine Dust-induced HaCaT keratinocytes, also downregulated inflammatory cytokines, tumour necrosis factor-α, interleukin (IL)-1β, -5, -6, -8, -13, interferon-γ, and chemokines, macrophage-derived chemokine, eotoxin, and thymus and activation regulated chemokine. Molecular mediators of MAPK and NF-κB pathway were downregulated by SHC4-6. This could successfully recover the impact of FD on skin barrier molecular mediators.	Fernando et al. (2021) [2]
LPS induced inflammation in macrophages is attenuated by fucoidan from *Sargassum swartzii*	RAW 264.7 macrophage cells	25–200 µg/mL	Fucoidan fraction F4 composed of Polysaccharide (approximately 60%), Sulfate (approximately 33.99%) with a low amount of Protein (0.41%) and Polyphenols (0.32 %)	Significantly decrease the NO production stimulated by LPS and also downregulate the expression of inflammatory mediators such as iNOS and COX-2 including pro-inflammatory cytokines (TNF-α, IL-6, and IL-1β), with a dose-dependent manner. The anti-inflammatory effect was exhibited via the suppression of TLR mediated MyD88, IKK complex ultimately blocking NF-κB and MAPK activation.	Jayawardena et al. (2020) [5]
Inflammatory responses stimulated via LPS in macrophages are inhibited via *Padina commersonii* purified fucoidan	RAW 264.7 macrophage cells	25–100 µg/mL	Purified fucoidan was rich in fucose and sulfate. Composed of 76.57 ± 2.54% polysaccharides and 11.20 ± 0.10% sulfates. FTIR results demonstrated structural similarity with commercial fucoidan.	Significantly down-regulated LPS-activated mRNA and protein expression levels of TLR2, TLR4, and MyD88 which are the inducers of NF-κB transcriptional factors via blocking TLR/MyD88/NF-κB signal transduction.	Sanjeewa et al. (2019) [39]
Anti-inflammatory effects of *Sargassum horneri* were evaluated in RAW 264.7 macrophages and zebrafish model	RAW 264.7 macrophage cells	12.5–50 µg/mL	A fucoidan (SHCF2) was purified via enzyme assisted extraction and FPLC system. Composed of polysaccharides (approximately 65%) and sulfate (approximately 12.5%) with protein (approximately 14%)	Inhibited the LPS-stimulated NO production in RAW 264.7 cells (IC50 = 40 μg/mL) via the nuclear factor-kappa B (NF-κB) and mitogen-activated protein kinase (MAPK) signal pathways. Specifically, SHCF2 down-regulated the heart-beating rate, cell death, ROS, and NO levels in LPS-exposed zebrafish embryo.	Sanjeewa et al. (2019) [62]
Potential molecular mechanisms of fucoidan from *Saccharina japonica* is evaluated against LPS induced macrophages	RAW 264.7 macrophage cells	50–200 µg/mL	Fraction 6 (SF6) Composed of polysaccharides approximately 58%) and sulfate (approximately 36%) with low amount of protein (approximately 1%)	SF6 remarkably inhibited LPS-induced production of various inflammatory mediators and pro-inflammation cytokines, including nitric oxide (NO), NO synthase (iNOS), cyclooxygenase-2 (COX-2), tumor necrosis factor-α (TNF-α), interleukin-β (IL-β), and interleukin-6 (IL-6). A mechanism study showed that SF6 could effectively inhibit inflammatory responses through blocking LPS-induced inflammation pathways, including nuclear factor-κB (NF-κB), mitogen-activated protein kinase (MAPK), and Janus kinase (JAK)-2 and signal transducer and activator of transcription (STAT)-1/3 pathways.	Ye et al. (2020) [70]

**Table 2:** Immunomodulatory potential of fucoidan purified from marine algae.

Study	Cell Line	Concentration	Algal Species and Fucoidan Purified	Cell Signaling Activity	Reference
Fucoidan altered the immunomodulatory markers and DCs phenotype	Human monocyte-derived dendritic cells (DCs)	100 μg/mL fucoidan	Standard fucoidan-Fucoidan purified from *Fucus vesiculosus* was purchased	Fucoidan elevated the expression of HLA-DR and co-stimulatory molecules of DCs, induces their Th1-promoting tumor necrosis factor α (TNF-α) and interleukin-12 (IL-12) secretion. This fucoidan is suggested to be used in DC-based vaccines for cancer immunotherapy.	Yang et al. (2008) [72]
Fucoidan express immunostimulating and DC maturing potential	Bone marrow-derived dendritic cells (DCs)	50 μg/mL	Standard fucoidan-Fucoidan purified from *Fucus vesiculosus* was purchased	The production of IL-12, TNF-α, major histocompatibility complex class I, II, CD54, and CD86 were promoted by fucoidan. Further fucoidan treated DCs expressed p65 (NF-κB) nuclear translocation.	Kim et al. (2008) [73]
Effect of fucoidan on spleen DCs and in vivo	Spleen dendritic cells (DCs)	C57BL/6 mice were treated with 10 mg/kg fucoidan for 24 h	Standard fucoidan-Fucoidan purified from *Fucus vesiculosus* was purchased	Systemic administration of fucoidan induced up-regulation of CD40, CD80 and CD86 expression and production of IL-6, IL-12 and TNF-a in spleen cDCs. Fucoidan also promoted the generation of IFN-c-producing Th1 and Tc1 cells in an IL-12-dependent manner. Moreover, fucoidan enhanced OVA-induced up-regulation of MHC class I and II on spleen cDCs and strongly prompted the proliferation of OVA-specific CD4 and CD8 T cells. The study reveals the potential of fucoidan to function as an adjuvant to induce Th1 immune response. Further, fucoidan promote CTL activation. Suggested to be useful in tumor vaccine development.	Jin et al. (2014) [74]
Immunomodulating potential of fucoidans on murine macrophages and splenocytes	RAW 264.7 cells, peritoneal macrophages and normal splenocytes	50–300 μg/mL	Anion exchange column purified fucoidan from *Undaria pinnatifida*	Fucoidan induced TNF-α expression from both types of macrophages. The TNF-α-inducing activity of UP-F was higher than that of FV-F. The chemokine expression (RANTES and MIP-1α) was also promoted in RAW 264.7 macrophages. The IL-6 including chemokines were significantly improved in UPF treated splenocytes.	Yoo et al. (2007) [75]
Macrophages and glial cells were examined for immune related properties against IFN-γ stimulation and fucoidan treatment	Glia (C6, BV-2) and macrophages (RAW 264.7, peritoneal primary cells)	50 μg/mL	Standard fucoidan-Fucoidan purified from *Fucus vesiculosus* was purchased	In glial cells IFN-γ induced inflammation was suppressed by fucoidan via JAK/STAT/IRF-1 and p-p38. The signaling positively regulated IFN-g-induced iNOS, which were inhibited by fucoidan. Contrastingly, in RAW macrophages, fucoidan promoted immune responses induced via IFN-γ. Confirmed the dual regulation of p38 in BV-2 microglia and primary peritoneal macrophages.	Do et al. (2010) [76]
In vivo viral replication and host immune defense system were assessed against fucoidan treatment	Macrophages were collected from BALB/c mice	10 μg/mL	Fucoidan prepared from *Undaria pinnatifida*	Fucoidan oral administration protected mice from infection with HSV-1. CTL activity of HSV-1 mice was enhanced by fucoidan. Phagocytic activity of macrophages and B cell blastogenesis in vitro was significantly stimulated by the fucoidan, while no significant change in the release of NO_2^−^_ by macrophages was observed.	Hayashi et al. (2008) [3]
Immune boosting properties of fucoidan from *Nizamuddinia zanardinii*	RAW 264.7 macrophage cell	10, 25 and 50 μg/mL	Anion exchange column purified fucoidan.	The active fraction (F3) promoted the secretion of NO, TNF-α, IL-1β, IL-6 in RAW 264.7 macrophages. Further NK cells were activated to release TNF-α, IFN-γ, granzyme-B, perforin, NKG2D and FasL. The activity was mediated through NF-κB and MAPK pathways.	Tabarsa et al. (2020) [100]

## 5. Conclusions

Brown algae are an approachable and substantial source of sulfated polysaccharides. Brown algal fucoidans have attracted attention as active ingredients for a wide array of medicinal applications. Some brown algal species are cultivated on a large scale and frequently consumed as food or food additives where occasional functional food and drug development interests are exhibited. This review article explores the structural diversity of fucoidans, extraction methodologies, and the associated impurities. Algal fucoidans have complicated heterogeneous structures. Despite the few reports on the structural elucidation of selected species, refined structures are not yet clear. Studies have been carried out with purified fucoidan, and most published research has been conducted with relatively crude fucoidan preparation, hindering the elucidation of the structure–activity relationship. Defined fucoidans and their conformational investigations could lead to a better understanding of this matter to fill the gaps. Chemical modifications in fucoidans, such as sulfation at predetermined positions, could considerably improve their respective bioactivities. Deeper studies on fucoidan structure will possibly facilitate the development and utilization of brown algal resources. Among the potent biological activities of fucoidans, this review delves into immunomodulatory and anti-inflammatory effects. Even though most research requires successful human trials, marine algal fucoidans conclusively provide therapeutic interventions to improve immunity and prevent immune disorders through cellular signaling. However, the number of clinical studies on this topic is limited, impeding knowledge of fucoidan pharmacokinetics. Consequently, this review aims to facilitate the awareness of brown algal fucoidans as a viable source of research material and a sustainable, utilizable material for industrial applications.
